# Sugarcane Cell Wall-Associated Defense Responses to Infection by *Sporisorium scitamineum*

**DOI:** 10.3389/fpls.2018.00698

**Published:** 2018-05-23

**Authors:** João P. R. Marques, Jeffrey W. Hoy, Beatriz Appezzato-da-Glória, Andrés F. G. Viveros, Maria L. C. Vieira, Niranjan Baisakh

**Affiliations:** ^1^School of Plant, Environmental, and Soil Sciences, Louisiana State University Agricultural Center, Baton Rouge, LA, United States; ^2^Genetics Department, Luiz de Queiroz College of Agriculture, University of São Paulo, Piracicaba, Brazil; ^3^Department of Plant Pathology and Crop Physiology, Louisiana State University Agricultural Center, Baton Rouge, LA, United States; ^4^Biological Science Department, Luiz de Queiroz College of Agriculture, University of São Paulo, Piracicaba, Brazil

**Keywords:** arabinoxylan, callose, histopathology, lignin, smut

## Abstract

The plant cell wall is known to be the first barrier against plant pathogens. Detailed information about sugarcane cell wall-associated defense responses to infection by the causal agent of smut, *Sporisorium scitamineum*, is scarce. Herein, (immuno)histochemical analysis of two smut resistant and two susceptible sugarcane cultivars was conducted to understand host cell wall structural and compositional modifications in response to fungal infection. Results showed that the fungus grew on the surface and infected the outermost bud scale of both susceptible and resistant cultivars. The present findings also supported the existence of early (24 h after inoculation) and later (72–96 h after inoculation) inducible histopathological responses related to the cell wall modification in resistant cultivars. Lignin and phenolic compounds accumulated during early stages of infection. Later infection response was characterized by the formation of a protective barrier layer with lignin, cellulose and arabinoxylan in the cell walls. Overall, the results suggest possible induction of cell wall-modified responses in smut resistant cultivars to prevent initial entry of the fungus into the meristematic tissues.

## Introduction

Sugarcane (*Saccharum* spp. hybrids) is a multifunctional crop plant. In addition to sucrose, its by-products generate impact in different sectors of the economy being used as food, fiber, fodder, plastic, and environmentally friendly fuel (Solomon, 2011). Sugarcane smut is a fungal disease that reduces productivity worldwide. The causal agent, *Sporisorium scitamineum* Syd. (Ustilaginomycetes), is a biotrophic fungus that can grow inter- or intracellularly causing damage to the host tissues (Marques et al., 2017). Many smuts affect floral tissues, but the one in sugarcane is a culm smut (Comstock, 2000). The fungus infects through germinating buds. The most recognizable symptom is the development of a whip-like sorus incited by the fungus at the shoot apex resulting from its association with the shoot apical meristem, which is influenced by the environment and host genotype. Anatomically, the sorus is an elongated internode, and the growth is mediated by high mitotic activity of the intercalary meristem at the base. The sorus produces billions of dark teliospores via thallic process, mostly restricted to the base of the sorus (Marques et al., 2017). Short and long-range dispersal is then accomplished by the aerial dissemination of teliospores. Breeding resistant cultivars is the most economical and environmentally sustainable method to control smut (Comstock, 2000; Magarey et al., 2012). Resistance to smut is a moderately heritable trait in sugarcane (Chao et al., 1990).

Sugarcane resistance to *S. scitamineum* infection is structural, biochemical and physiological, and involves both constitutive and inducible responses (Appezzato-da-Glória et al., 1995; De Armas et al., 2007; Santiago et al., 2010a). Appezzato-da-Glória et al. (1995) presented the first evidence that resistant cultivars exhibited structural features against smut fungal infection. Resistant cultivar SP70-1143 possessed a higher number of bud scales with lignified cells, phenolic compounds accumulated in the epidermis, and a higher number of trichomes were present as compared to susceptible cultivar NA56-79. Biochemically, glycosidic compounds within the tissues can inhibit teliospore germination (Millanes et al., 2005). Also, sugarcane tissues infected with *S. scitamineum* showed an increase in pH and thiol (SH) polyamines and of phenolic compounds, such as caffeic, syringic, *p-*coumaric, ferulic and *p-*hydroxybenzoic acids (De Armas et al., 2007). The increased level of different polyamines and their possible combination with phenols in mature plant tissues being attacked by the fungus indicates another potential mechanism of resistance (Piñon et al., 1999). Enzymes related to reactive oxygen species (ROS)-scavenging, such as peroxidase, ascorbate peroxidase, catalase and superoxide dismutase, were observed in higher concentration in resistant sugarcane cultivars suggesting their possible utility as markers in resistance screening (Su et al., 2016). Pathogenesis related (PR)-proteins, chitinase and β-1,3 glucanase, also showed increased expression in some resistant cultivars (Su et al., 2015, 2016).

The plant cell wall plays a vital role as a physical and chemical barrier, potentially conferring immunity, against different plant pathogens (Albersheim et al., 2011; Malinovsky et al., 2014). The composition and function of the cell wall varies in different plant tissues or organs. Cellulose (β-1,4-linked glucan chains), the major component of the cell wall, is arranged in microfibrils that are immersed in an amorphous polysaccharide matrix composed mainly of pectins and hemicelluloses. The hemicellulose composition among the flowering plants is different where eudicots have Type I cell walls with xyloglucan abundance while Poaceae have Type II cell walls constituted by arabinoxylan (Carpita and Gibeaut, 1993; Albersheim et al., 2011). In sugarcane, arabinoxylan was suggested to be strongly bound to cellulose (Souza et al., 2013). Recently, the first evidence of arabinoxylan distribution in sugarcane stems was demonstrated by immuno-histochemistry using LM11 antibody (Costa et al., 2016). The ground parenchyma and the lignified cells surrounding the vascular bundles can present arabinoxylan in their walls. The last fraction of the sugarcane cell wall is composed of pectins at about 10% (Souza et al., 2013).

Both pre- and post-infection mechanisms of smut defense were suggested (Lloyd and Pillay, 1980). The authors suggested that the post-infection defense response involves a cell-wall mechanism. Callose is by far the most studied carbohydrate polymer related to cell wall defense. Callose depositions in the cell wall are an important feature of immunity, and deposition is commonly triggered by microbe- or pathogen-associated molecular patterns (MAMPs or PAMPs) (Jones and Dangl, 2006; Li et al., 2016). Sugarcane cells of the sorus are able to deposit callose at *S. scitamineum* sites of penetration or surrounding its intracellular hyphae, indicating a possible role of callose in the structural defense response (Marques et al., 2017). Callose was also observed in the sieve plate pores throughout the phloem of the sorus. In addition to callose, other classes of compounds, such as lignin, phenolic-polyamines, peroxidases, arabinogalactan hydroxyproline-rich glycoproteins, pectin, xyloglucan, arabinoxylan, and cellulose have been implicated in plant cell wall defense (Underwood, 2012; Chowdhury et al., 2014). However, no information is available to illustrate the cell-wall responses in the beginning of the compatible and incompatible sugarcane-smut interactions.

Secondary cell walls exhibit a deposition of lignin that is composed of a heterogeneous hydrophobic polymer of phenylpropanoid units (Campbell and Sederoff, 1996). Lignin composition varies in radial and axial directions throughout the sugarcane culm (Bottcher et al., 2013). Lignin also contributes to cell wall recalcitrance and acts as an important structural barrier to pathogen infection (Malinovsky et al., 2014) that can be associated with papillae in some incompatible pathosystems (Underwood, 2012). During abiotic and biotic stress, plant cells enhance lignin biosynthesis through the phenylpropanoid pathway, which is also responsible for the production of several other phenolic compounds related to plant defense (Malinovsky et al., 2014). Sugarcane cell wall-associated responses were observed in leaves inoculated with *S. scitamineum* elicitors extracted from teliospores (Santiago et al., 2010a). The thickness of the lignified cell walls of sclereids and xylem vessels in leaves was increased. Inoculation with the fungus also leads to increased levels of hydroxycinnamic acids and their derivatives, which enhances the synthesis of lignin, strengthening the cell walls of resistant cultivars (Santiago et al., 2010a). Recently, it was also observed that shikimate hydroxycinnamoyl transferase, cinnamoyl-CoA reductase, and peroxidase were up-regulated in the sorus indicating possible elicitation of lignin biosynthesis pathway by *S. scitamineum* (Schaker et al., 2016). Infected resistant meristems also presented an increase of phenylalanine ammonia lyase activity (De Armas et al., 2007; Barnabas et al., 2016). Lignin polymerization strengthening the challenged cell walls also requires H_2_O_2_ (Hatfield and Vermerris, 2001; Huckelhoven, 2007). ROS are well-documented biotic stress signaling molecules and have a relationship with papillae formation (Luna et al., 2011; Faulkner, 2015).

Based on the importance of the cell wall in plant defense responses, it is essential to study its composition in compatible and non-compatible sugarcane-smut interactions at the beginning of the infection process. The present study was undertaken to compare cell wall modification in two resistant and two smut susceptible sugarcane cultivars with an objective to determine their effects on *S. scitamineum* initial infection and colonization.

## Materials and Methods

### Plant Material

Single-node cuttings (150 for each cultivar) were collected from the intermediate nodes of 5-month-old plants of two smut resistant sugarcane cultivars LCP 85-384 and HoCP 96-540 and two susceptible cultivars L 99-226 and L 01-299. The buds were initially subjected to a hot water treatment (52°C, 30 min) followed by surface sterilization in a 0.01% sodium hypochlorite solution (10 min) and subsequently washed three times in sterile distilled water. The cuttings were then placed with buds facing up in sterilized 32 cell-styrofoam trays containing autoclaved vermiculite.

### Inoculation Procedure

Prior to inoculation, a viability test was carried out by incubating a 100-μL teliospore suspension (1 × 10^4^ teliospores mL^-1^) on 0.1% agar-water medium (28°C, 8 h). The percentage of germination was estimated by counting germination in multiples of 100 teliospores on the agar surface, and inoculum with a germination rate higher than 90% was used. Inoculation was performed by placing 20 μl of the fungal suspension (10^5^ spore mL^-1^ of water in 0.01% Tween^®^ 20) on the top of sugarcane buds (outermost scale). Seventy-five buds of each cultivar were inoculated with the fungus. As a control, 75 buds of each cultivar were inoculated with aqueous solution containing 0.01% Tween^®^ 20. After the inoculation, the buds were kept inside a growth chamber at 27°C in the dark.

### Microscopic Analysis

To better understand defense responses associated with the composition of the cell wall, histochemical tests were conducted with buds 24, 48, 72, and 96 h after inoculation (h.a.i.). They were fixed in FAA solution (formaldehyde, glacial acetic acid and ethanol 70%; 5:5:90), subjected to vacuum (two times 15 min each) for 24 h, hydrated in decreasing ethanol gradient (50, 30, 10%), and transferred to a PBS solution (0.2 M Na_2_HPO_4_ and 0.2 M NaH_2_PO_4_; pH 7.2) for overnight at 4°C.

The frozen tissue sectioning was performed following Nakazono et al. (2003). Firstly, the sugarcane buds were transferred to a 15 ml tube with 10% sucrose for 1 day under vacuum followed by centrifugation 2 × 3.500 rpm for 15 min each and then 15% sucrose for 3–4 days under vacuum (six times – 15 min each) and centrifuging 8 × 3.500 rpm for 20 min each. Buds were then stored at 4°C. Buds were then infiltrated in Tissue-Tek optimal cutting temperature (OCT) compound (Sakura Finitek, Torrance, CA, United States) in Tissue-Tek cryomolds 10 mm × 10 mm × 5 mm. After adjusting the orientation, the samples were immediately frozen by floating the cryomolds in liquid nitrogen. The blocks were stored at -80°C for 1 h and then transferred to -20°C overnight to equilibrate the temperature. The OCT blocks were longitudinally sectioned (10–12 μm of thickness) using a Leica 3050 Cryostat (Leica Biosystems, Buffalo Grove, IL, United States) at -25°C and the sections were collected on CryoJane transfer adhesive tape (Leica Biosystems). The sections were then transferred to 1× coated slides (Leica Biosystems) and fixed by exposing to a flash of UV light. Fungal cell wall structure was visualized by staining the slides with WGA-AF 488 (Life Technologies, Carlsbad, CA, United States) and analyzing under TC/GFP Filter (ex: 470/40; em: 525/50), according to the manufacturer’s instructions. Aniline blue 0.05% in PBS (pH 8.4) was used to stain the callose (Ruzin, 1999) that was analyzed under epifluoresce microscopy with A4 filter (ex: 340-380 nm; em: 450-490 nm). Cellulose visualization was done by 1% Calcofluor White under fluorescence using a DAPI filter. Arabinoxylan was probed with LM11 rat primary antibody (PlantProbes, Leeds, United Kingdom) for 3 h and then detected using a goat secondary antibody anti-rat IgG attached to Texas Red for 3 h and observed under TXRED filter (Ex 490/20; Em: BP 645/75). Lignin was stained with 0.1% phloroglucinol (Ruzin, 1999), and phenolic compounds were visualized after treating the sections with 5% ferric chloride (Ruzin, 1999). Both were then analyzed under light microcopy. Double staining methods were used to localize the main cell wall components together with *S. scitamineum* hyphae. Thus, the immunolabeled slides with LM11, Calcofluor White and ferric chloride were counterstained with WGA-AF488. All staining steps were carried out at room temperature.

Images from the slides were captured digitally through an epifluorescence microscope Leica DM6000B. The images were captured using a QE-enhanced ORCA-Flash4.0 V2 with 82% peak QE (Hamamatsu Photonics, Bridgewater, NJ, United States) and processed with LAS-X Software (Leica Biosystems). Deconvolution was applied when necessary with a calculated point-spread function. Cellulose and arabinoxylan accumulations were quantified from 20 cells using Image J^1^ with the formula: corrected total cell fluorescence = integrated density – (area of selected cell × mean fluorescence of background readings).

### Scanning Electron Microscopy (SEM)

Scanning electron microscopy (SEM) was carried out to observe the developmental steps of *S. scitamineum* on inoculated sugarcane buds. Sugarcane buds were harvested at 12, 24, and 48 h.a.i. and immediately fixed in FAA solution. Shaking was avoided during the fixation process to avoid disturbance to the fungal structures on the bud scale surface. The samples were dehydrated in an ethanol series and by critical point drying (Horridge and Tamm, 1969). The dried samples were glued on aluminum stubs, coated with platinum and examined with a JSM -6610 LV SEM (Jeol USA, Peabody, MA, United States) at 10 kV.

## Results

### Anatomical Features of Outermost Bud Scales

**FIGURE 1:**
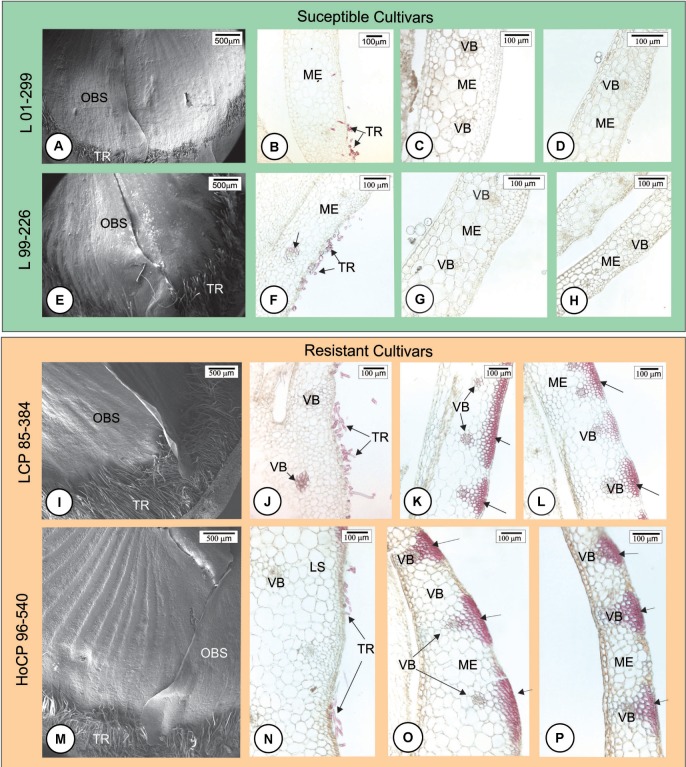
Anatomical features of the outermost scale of four Louisiana sugarcane cultivars 24 h after mock inoculation (without fungus) with Tween^®^ 20 0.01% in distilled water. **(A,E,I,M)** Scanning electron micrographs. **(B–D,F–H,J–L,N–P)** Light micrographs after phloroglucin histochemical test. Longitudinal sections of the base **(B,F,J,N)**, middle **(C,G,K,O)** and apex **(D,H,L,P)** of outermost bud scale. **(A–H)** Susceptible cultivars. Note that there are few trichomes (TR) at the base of the bud especially in L 01-299. After phloroglucin test, lignin staining was not observed throughout the outermost scale. Lignified cell walls are present only in trichome and tracheary elements (arrow in **F**). **(I–P)** Resistant cultivars. **(I,M)** Lignified trichomes were more numerous at bud scale base. **(I,J,M,N)** Lignin staining occurred in cell walls of trichomes, tracheary elements, bundle sheath extensions and exterior epidermal cells where vascular bundles are located (arrows) at the middle **(K,O)** and apex **(L,P)** of outermost scale but not at its base **(J,N)**. ME, mesophyll; OBS, outermost bud scale; TR, trichome; VB, vascular bundle.

Anatomical features of the healthy outermost bud scales of resistant (LCP 85-384 and HoCP 96-540) and susceptible (L 01-299 and L 99-226) cultivars were observed 24 h.a.i. with Tween^®^ 20 0.01% in distilled water (mock inoculation control). All cultivars presented two types of non-glandular trichomes: a two-celled non-lignified trichome (Supplementary Figure 1) and a one-celled thickened lignified trichome (**Figures 1A,B,E,F,I,J,M,N**). The lignified ones were mainly present at the base of the bud scale, but they also were observed at the margin while the non-lignified occurred close to the bud scale margins (Supplementary Figure 1). Buds of resistant cultivars had more trichomes than susceptible ones (**Figures 1A,E,I,M**), especially in ‘LCP 85-384’ that has numerous longer trichomes at the base of the scale (**Figure 1I**). Lignin was observed in different cell types along the surface of the outermost bud scale. Both susceptible cultivars showed lignified cell walls in trichomes (**Figures 1B–D,F–H**) and in tracheary elements in the base of the bud scale (**Figure 1F**). In contrast, resistant cultivars showed lignified cell walls in the epidermal cells as well as in the cells of the bundle sheath extensions facing the adaxial side at the middle (**Figures 1K,O**) and apex (**Figures 1L,P**) of the outermost bud scale but not at its base (**Figures 1J,N**). Comparing the resistant cultivars (**Figures 1K,L,O,P**), lignin deposition in LCP 85-384 was observed in the parenchyma cells between vascular bundles in addition to the epidermal cells. All cultivars exhibited lignin deposition in the cell walls of epidermis and tracheary elements in the outermost bud scale after 48 h of mock inoculation with Tween^®^ 20 0.01% in distilled water.

### Fungal Infection of Sugarcane Buds

**FIGURE 2:**
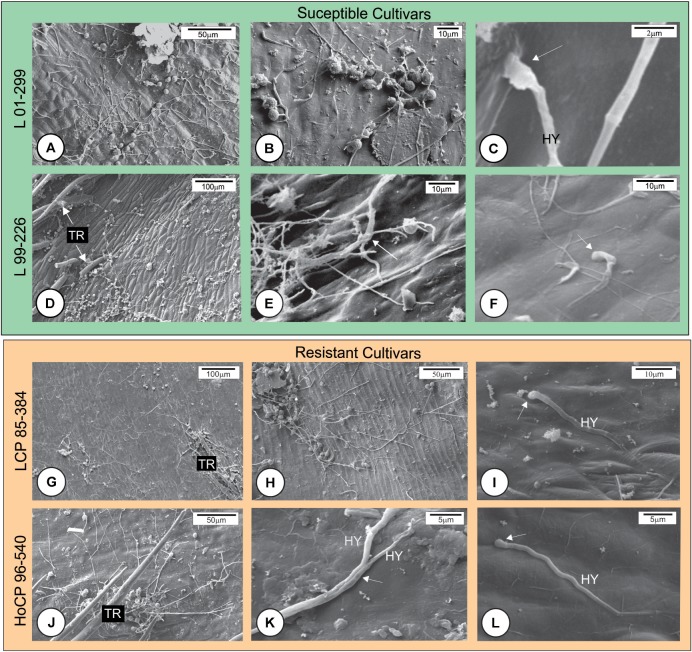
Scanning electron micrographs of the initial development of *Sporisorium scitamineum* on the surface of the outermost bud scale of four Louisiana sugarcane cultivars. **(A–F)** Susceptible cultivars. **(G–L)** Resistant cultivars. **(A,D,G,J)** Fungal growth at the base of outermost bud scales. Note that the fungus grew vigorously on all cultivars. No apressorium-like structures were observed at 12 h after inoculation (h.a.i.). **(B,E,H,K)** At 24 h.a.i., an increase in fungal growth was observed, and hyphae were observed in physical contact with each other (arrows) **(E,K)**. **(C,F,I,L)** Apressorium-like structures (arrows) were observed on all cultivars at 24 h.a.i. HY, hyphae; TR, trichome.

**FIGURE 3:**
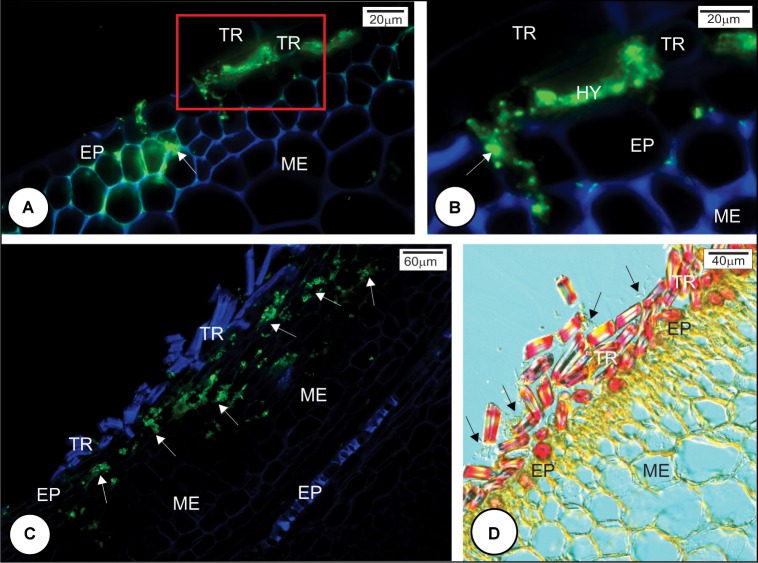
*Sporisorium scitamineum* penetration in resistant and susceptible sugarcane cultivar outermost bud scales at 24 h after inoculation. **(A,B)** L 99-226 susceptible cultivar; **(C,D)** LCP 85-384 resistant cultivar. **(A–C)** *S. scitamineum* hyphae stained green with WGA Alexa Fluor 488, and the plant cell wall stained blue with Calcofluor White. **(D)** Differential interference contrast micrography after phloroglucin reaction. **(A,C)** Fungal hyphae (arrows) were observed in both cultivars at the base of the scale under the trichomes. **(B)** Detail (100×) of the rectangle in **(A)**; the fungus enters by the anticlinal walls of epidermis (arrow). **(C)** Fungal hyphae in the bud scale mesophyll (arrows). **(D)** Resistant cultivar LCP 85-384 exhibiting lignin (pink color) just in trichome cell walls but not in subjacent epidermal cell walls at the base of outermost bud scale. Note the abundance of *S. scitamineum* hyphae among trichomes (arrows). EP, epidermis; HY, hyphae; ME, mesophyll; TR, trichome.

Scanning electron microscopy analysis indicated that the fungal teliospores germinated and the pro-mycelia grew on the surface of the outermost bud scale, but because the sugarcane bud is not a flat structure, the drop of fungal spore suspension, when placed on the top of the bud, ran down to the base where numerous trichomes occur. Teliospore germination and pro-mycelial growth occurred in this region within 12 h.a.i. in all cultivars (**Figures 2A,D,G,J**). Between 12 and 24 h, hyphal fusion occurred in the fungal pro-mycelia, and the hyphae grew vigorously on the surface of resistant and susceptible cultivars (**Figures 2B,H**). The preponderant characteristic observed at this time interval was the anastomosis of the pro-mycelia when the fungal structures touched each other (**Figures 2E,K**). Subsequently, apressorium-like structures developed preferentially on the anticlinal cell walls of the outermost bud scale epidermis (**Figures 2C,F,I,L**). Apressorium over the periclinal cell walls was not observed. Staining of the cross section of the outermost bud scale with WGA-AF488 and Calcofluor White revealed that *S. scitamineum* had already penetrated and colonized mesophyll cells of both resistant and susceptible cultivars at 24 h.a.i. (**Figures 3A–C**). Infection occurred mainly at the base of the outermost scale where the mesophyll and epidermal cells are not lignified in both resistant and susceptible cultivars (**Figure 3D**). In both cases, *S. scitamineum* penetrated through an intercellular pathway along the anticlinal cell walls of the epidermis (**Figure 3B**). After its entrance, the fungus grew in the mesophyll both inter- and intracellularly.

### Histopathological Sugarcane Responses Against *S. scitamineum*

The study focused on elucidating both early and later responses of sugarcane bud scale tissues to *S. scitamineum* infection. Infection events were distinguished at 24 and 48 h.a.i. (initial prevention of infection) and between 72 and 96 h.a.i. (prevention of colonization in the meristematic tissues).

**FIGURE 4:**
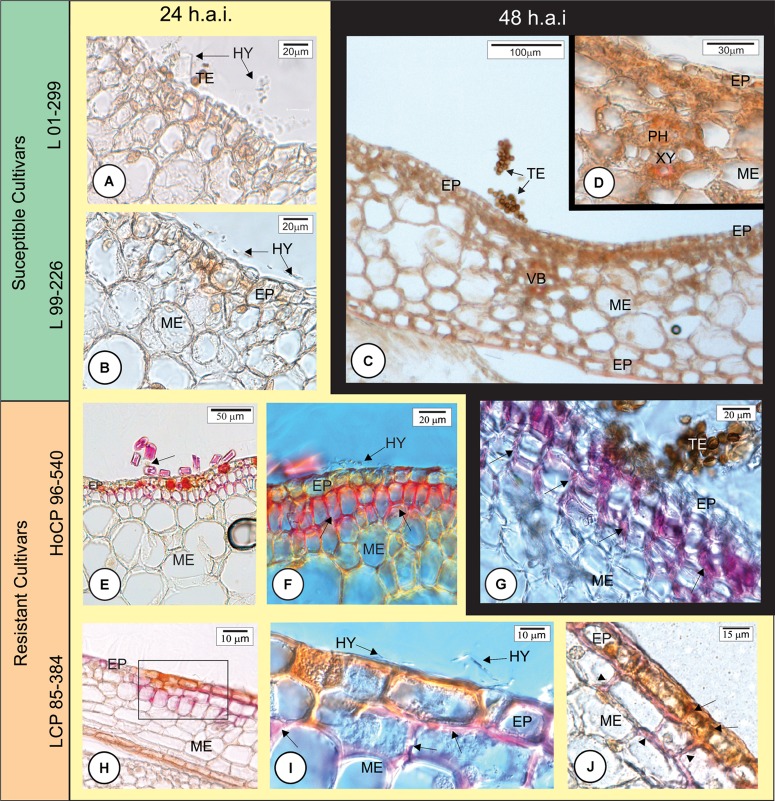
Early anatomical responses of Louisiana sugarcane cultivars to *Sporisorium scitamineum* infection. The photomicrographs in horizontal axis are for different cultivars. Background yellow – 24 h after inoculation (h.a.i.); black – 48 h.a.i. **(A–D)** Susceptible cultivars. **(E–J)** Resistant cultivars. **(A–I)** Histochemical test with phloroglucin to detect lignin (pink color). **(J)** Double histochemical test using phloroglucin for lignin (pink) and ferric chloride to detect phenolic compounds (brown color). **(A–D)** All inoculated susceptible cultivars did not exhibit lignin deposition in the cell walls beneath the fungal infection site at 24 **(A,B)** or 48 h.a.i. for cultivar L 99-226 **(C,D)**. **(E–I)** Both resistant cultivars showed lignin deposition in the periclinal and anticlinal walls of epidermal and mesophyll cells in close proximity to *S. scitamineum* hyphae (arrows in **F,G,I**). Arrow in **(E)** indicates a fugal hypha among trichomes. **(I)** Detail of the square in **(H)** showing fungal hyphae and lignified mesophyll parenchyma cell walls. **(J)** Epidermal cells exhibiting accumulated phenolic compounds (arrows) and parenchyma cells exhibiting lignification of the cell walls (arrowheads). EP, epidermis; HY, hyphae; PH, phloem; ME, mesophyll; TE, teliospore; VB, vascular bundle; XY, xylem.

At 24 and 48 h.a.i., susceptible cultivars did not exhibit lignin deposition in the challenged plant cells underlying the fungal hyphae infection sites (**Figures 4A–D**, **8B**). In contrast, in both resistant cultivars, lignin was observed in epidermal and parenchyma cell walls of the outermost bud scale in close proximity to *S. scitamineum* hyphae (**Figures 4E–J**, **8A,C**). Furthermore, epidermal cells in contact with the fungus accumulated phenolic compounds (**Figures 4I**, **8A,C**) in both resistant cultivars. It is important to note that fungus infection was not observed where the cells had lignin and phenolic compounds in their walls. In susceptible cultivar tissues, no lignin or phenolic compound accumulation was observed while comparing inoculated with non-inoculated bud scales.

**FIGURE 5:**
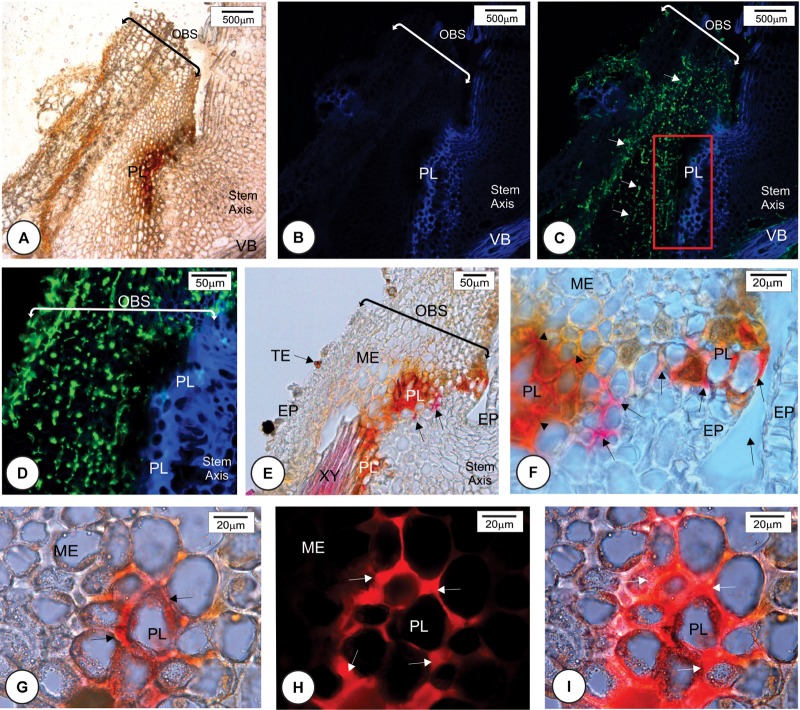
Later anatomical responses of resistant cultivar HoCP 96-540 to *Sporisorium scitamineum* infection at 72 h after inoculation. **(A,E,F)** Bright field images. **(B–D,H,I)** Fluorescent images. **(A–C)** Insertion point of the bud scale to the stem axis. **(A)** Bright field. **(B)** Calcofluor White histochemical test. **(C)** Merged image after double staining method with Calcofluor White and WGA Alexa Fluor 488. The fungus in green (arrows) was observed at the base of bud scale but was not observed to enter the stem axis because of the development of a protective barrier layer (PL). **(D–F)** Details of the rectangle in **C**. **(D)** Note that the fungus in green did not penetrate the protective layer. **(E,F)** Phloroglucin histochemical test indicating lignin deposition in several cell walls of mesophyll parenchyma and epidermis (full arrows). The protective layer also showed some cells that present a natural reddish color (arrowheads in **F**). **(G,I)** Immunohistochemical test using a primary arabinoxylan antibody LM11 and a second antibody conjugated with Texas Red. **(G)** Bright field micrograph showing a natural reddish color parenchyma cell walls (arrows). **(H)** Same region after immunohistochemical test with LM11 showing arabinoxylan deposition in some cell walls (arrows). **(I)** Merged image. EP, epidermis; HY, fungal hyphae; OBS, outermost bud scale; ME, mesophyll; PA, parenchyma; TE, teliospore; VB, vascular bundle; XY, xylem.

**FIGURE 6:**
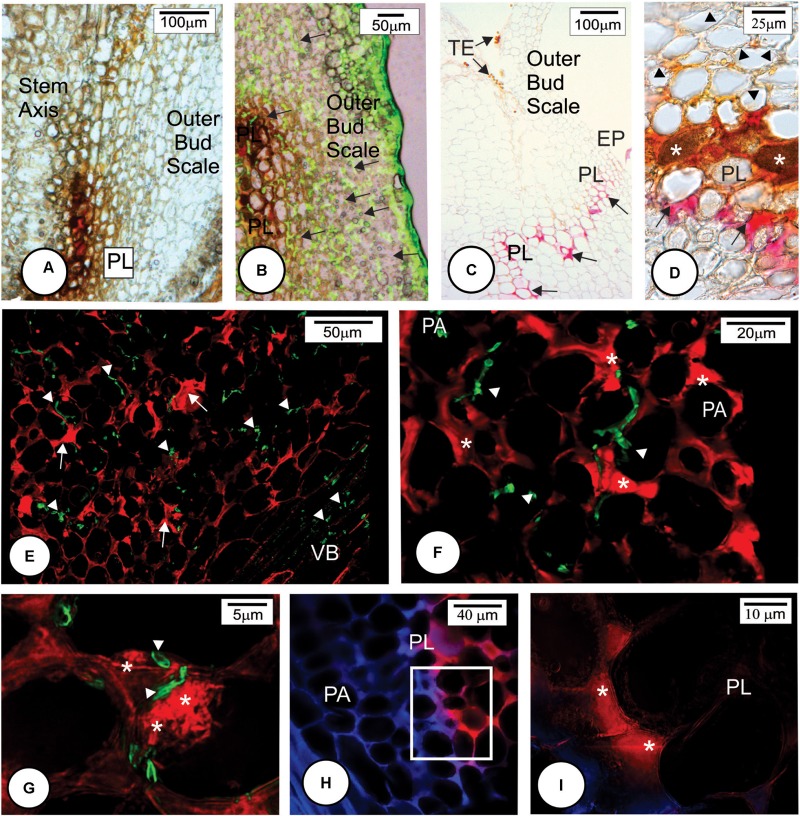
Later anatomical responses of resistant cultivar LCP 85-384 to *Sporisorium scitamineum* infection at 96 h after inoculation. **(A–C)** Bright field images. **(B,D–G)** Fluorescent images. **(A)** Protective layer cells exhibited accumulations of phenolic compounds (brown color). **(B)** Merged image of bright field showing phenolic compounds in brown and fluorescent image exhibiting the fungus in green color (arrows). **(C)** Phloroglucin histochemical test. Lignin (pink color) was observed in anticlinal and periclinal walls of parenchyma cells forming a protective layer (arrows). **(D)** Phloroglucin histochemical test after ferric chloride reaction. Phenolic compounds (^∗^) accumulated in non-lignified cell walls. Lignified cell walls (arrows). Note that the cells colonized by fungus have cell wall modification (arrowhead). **(E–G)** Double staining with LM11 detecting arabinoxylan (arrows) and WGA Alexa Fluor 488 detecting fungal hyphae (arrowheads) in the colonized outermost bud scale. Note arabinoxylan deposition in some cell walls close to the fungal infection site (^∗^ in **F**,**G**). The fungal hyphae stained green (arrowheads). **(H,I)** Double staining with LM11 and Calcofluor White in the protective layer periphery. The outermost bud scale is located to the right. **(H)** Detail of square in **(G)** showing high arabinoxylan deposition in protective layer cells. EP, epidermis; ME, mesophyll; PA, parenchyma; PL, protective layer; TE, teliospore; VB, vascular bundle.

The fungus successfully infected all cultivars but mainly at the base of the outermost bud scale where the mesophyll cells were not lignified and did not accumulate phenolic compounds in their walls (**Figure 3D**). Once infected and colonized, the fungus grew toward the point of outermost scale insertion in the stem axis where a barrier layer with different cell wall composition was observed at 72 h.a.i. in resistant cultivar HoCP 96-540 and at 72–96 h.a.i. in resistant cultivar LCP 85-384 (**Figures 5**, **6**). In both resistant cultivars, *S. scitamineum* hyphae did not pass through the protective barrier layer (**Figures 5C,D**, **6B**).

Multiple histochemical tests detected different wall components of the protective barrier layer cells. Lignin was a major component (**Figures 5E,F**, **6C,D**) that was deposited in periclinal and anticlinal cell walls across the mesophyll cells (**Figures 5F**, **6C**). In some regions, there was a high deposition of lignin in the intercellular spaces (**Figure 6C**). Phenolic compounds were also associated with the protective layer (**Figures 6A,B**). Phenolic compounds were observed to be accumulated inside non-lignified cells (**Figure 6D**). When non-stained tissues from HoCP 96-540 at 72 h.a.i. were observed under bright field, reddish cell walls were observed in the protective layer (**Figures 5G–I**), and immunohistochemistry assay with LM11 antibody indicated accumulation of arabinoxylan in the cell walls (**Figures 5H,I** and Supplementary Figure 3). Arabinoxylan accumulation was detected in cell walls of LCP 85-384 infected cells at 96 h.a.i. (**Figures 6C–G**). Phenolic compounds were also accumulated inside the cells with arabinoxylan-rich walls (**Figures 6D**, **8D,F**). In some regions at the base of the outermost bud scale, arabinoxylan accumulation was observed (**Figures 6E,F**), and these accumulations appeared to be associated with fungal infection sites (**Figure 6G**).

**FIGURE 7:**
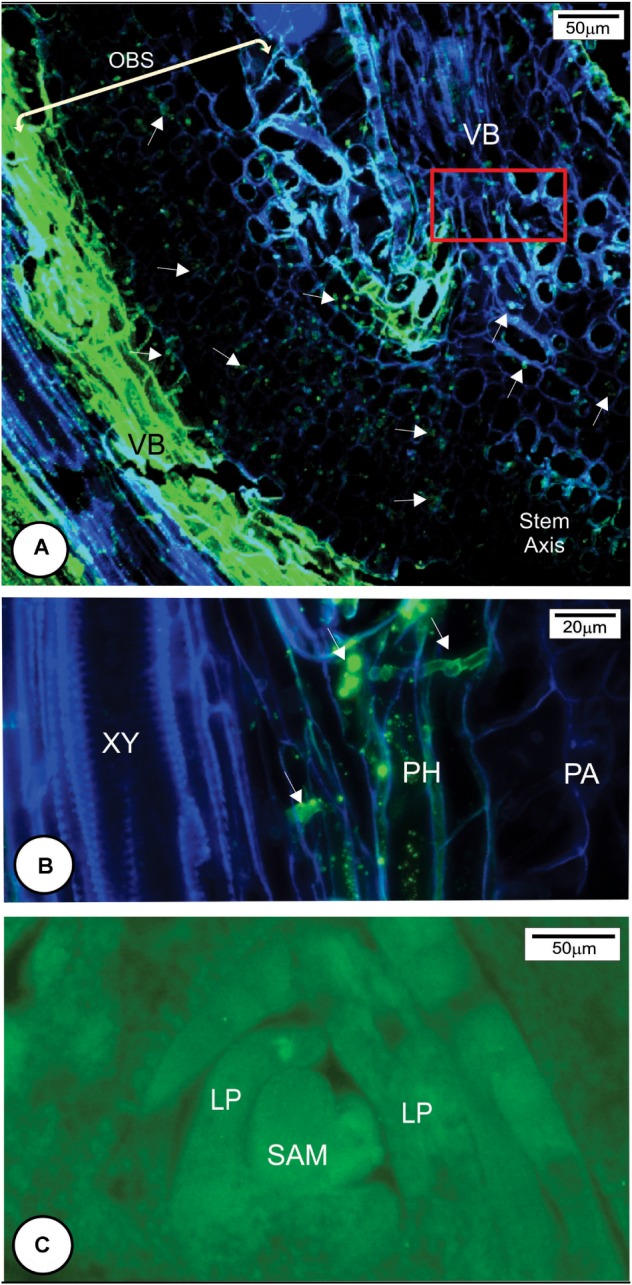
*Sporisorium scitamineum* colonization in susceptible cultivar L 99-226 at 96 h after inoculation. **(A)** Overview of the fungal hyphae infection route (arrows) to reach the stem axis (in the right of the image) through the outermost bud scale base. **(B)** Detail of the rectangle in **(A)**. Note that the fungal hyphae colonize phloem cells. **(C)** Overview of the shoot apical meristem and leaf primordia stained with WGA Alexa Fluor 488. Note the absence of *S. scitamineum* hyphae in the shoot apical meristem. LP, leaf primordium; PA, parenchyma; PH, phloem; SAM, shoot apical meristem; VB, vascular bundle; XY, xylem.

**FIGURE 8:**
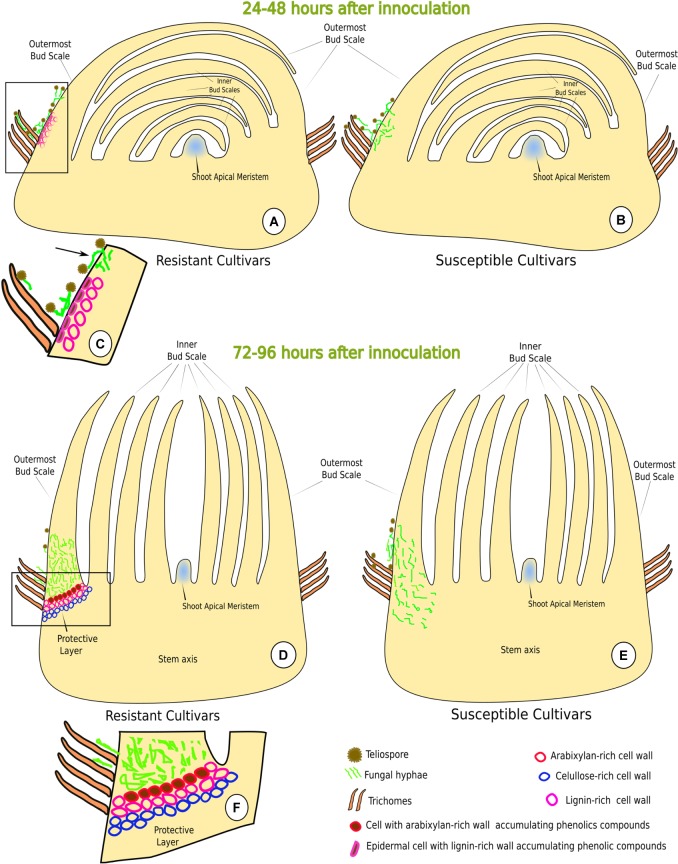
Schematic model depicting sugarcane-*Sporisorium scitamineum* interaction in resistant (LCP 85-384 and HoCP 96-540) and susceptible (L 01-299 and L 99-226) varieties. **(A–C)** Early stages of interaction (24–48 h.a.i.). **(C)** Detail (rectangle in **A**) of the early resistance response, i.e., epidermal cells with lignified walls and phenols accumulation at the base of the outermost bud scale. Note that the fungus cannot penetrate through the lignified regions of the bud scale but can penetrate at non-lignified regions (arrow). **(B)** The susceptible cultivars do not exhibit any structural response. **(D–F)** Late stages of the interaction (72–96 h.a.i.). **(E)** During late stage, the fungus was not able to reach the shoot apical meristem. **(F)** Detail (rectangle in **D**) of the protective layer where cell walls have accumulation of lignin, arabinoxylan and cellulose.

Callose (Supplementary Figure 2) and cellulose were also associated with the protective layer (**Figures 5B,D**, **6H**). Cellulose occurs naturally in all cell walls but seemed to accumulate more in the thickened cell walls of the protective layer that prevented the fungus from reaching the meristematic tissues (**Figures 5I**, **8D,F** and Supplementary Figure 3). One interesting aspect of the protective layer was that it was difficult to detect cellulose in cell walls with a high concentration of arabinoxylan and vice versa. This suggested there was a gradient of cell wall composition between cellulosic cell walls to arabinoxylan-rich walls (**Figures 6H,I**). A protective layer was not observed in infected susceptible cultivars (**Figures 7A–C**, **8E**), and *S. scitamineum* was able to colonize the vascular bundles of the stem axis at 96 h.a.i. (**Figures 7A,B**) but without reaching the shoot apical meristem at this time (**Figures 7C**, **8E**).

## Discussion

Sugarcane genotypes differed in their resistance reaction against *S. scitamineum* based on the morphological characteristics of the buds and in response to different inoculation methods (Waller, 1970). A resistant cultivar was shown to exhibit a higher number of bud scales with epidermal cells with lignified walls and phenolic compounds accumulated in the epidermis, and trichome density was higher in the resistant cultivar compared to the susceptible one (Appezzato-da-Glória et al., 1995). In the present work, two resistant cultivars HoCP 96-540 and LCP 85-384 presented similar constitutive barriers that were absent in two susceptible ones. Susceptible cultivars presented fewer trichomes, and no lignin was observed in the walls of epidermis and bundle sheath extensions cells. It is important to note that LCP 85-384 is one of the parents of HoCP 96-540 and L 01-299, but shares 54% and 48% genetic similarity with them, respectively (Parco et al., 2011). On the other hand, both susceptible cultivars are 42% similar genetically.

It is noteworthy to mention that the fungus successfully infected all cultivars but mainly at the base of the outermost bud scale where the cells in the resistant cultivars were not lignified. These results indicated that lignin could act as a local barrier as has been reported by other authors (Appezzato-da-Glória et al., 1995; Legaz et al., 2011).

In this study, trichomes were found more abundant on bud scales of resistant cultivars as observed in a resistant cultivar by Appezzato-da-Glória et al. (1995), but abundant teliospore germination and hyphal growth were observed at the base of the outermost bud scale even in the resistant cultivars. This contradicts the previous observation by Appezzato-da-Glória et al. (1995) that these trichomes could play a role in the prevention of infection by spore adhesion at the trichomes exterior.

### Fungal Penetration and Colonization

The first report of sugarcane smut histopathology by Alexander and Ramakrishnan (1980) showed that *S. scitamineum* did not infect the bud scales but grew on the surface of the scales and at the base of innermost scale. In this study, the fungus effectively infected and colonized the outermost scales of both resistant and susceptible cultivars. The authors described fungal entry into the meristem between 6 and 36 h after teliospores were deposited on the bud. However, they did not show any evidence of the infection or colonization process. Our results showed that *S. scitamineum* teliospores were germinating and promycelia forming at 12 h.a.i. Between 12 and 24 h.a.i., *S. scitamineum* promycelia were observed in contact with one another resulting in plasmogamy and consequent reciprocal nuclear exchange to establish dikaryotic infection hyphae. In addition, the formation of an inflated apressorium-like structure was observed on the anticlinal cell wall of epidermis in all cultivars. The apressorium-like structure could be responsible for penetration of the cuticle and epidermal cell wall in order to infect the outermost bud scale. This structure was preferentially associated with the epidermal anticlinal cell wall suggesting that the fungus possibly uses the topographical information for thigmotropic growth to follow the interstices between cells in order to facilitate infection. The penetration between epidermal cells was also detected in several other pathosystems, including *Peronospora parasitica × Brassica oleracea* and *Erysiphe polygoni × Trifolium pratense* (Preece et al., 1967), *Ustilago maydis × Zea mays* (Snetselaar and Mims, 1994) and *Colletotrichum acutatum × Citrus sinensis* (Marques et al., 2013).

Previous literature suggested that *S. scitamineum* did not infect sugarcane bud scales (Singh and Budhraja, 1964; Alexander and Ramakrishnan, 1980), but stained sections of the outermost bud scale in our study showed that the fungus entered the base of the scale. The fungus penetrated through epidermis anticlinal cell walls and afterward the colonization was both intra- and intercellular. Marques et al. (2017) demonstrated that *S. scitamineum* is able to degrade the middle lamellae and change the crystalline cellulose arrangement to promote intercellular growth and then penetrates the protoplasm without breaking the plasma membrane. In this study, after colonization of the outermost scale of the susceptible cultivars, the fungus entered the stem axis colonizing the ground and vascular tissues. The colonization of vascular tissues also was reported by others (Lloyd and Pillay, 1980; Marques et al., 2017), and this would provide a path to reach the meristematic tissues. After penetration and consequent colonization of the outermost bud scale, the fungus entered the internal tissues only in susceptible cultivars, but infection was not observed to reach the shoot apical meristem until 96 h.a.i. (**Figure 8E**). However, Alexander and Ramakrishnan (1980) reported that the fungus infected the meristematic tissues between 6 and 36 h.a.i. after smearing the sugarcane with ustilospores. Differences in the time required for *S. scitamineum* to reach the shoot meristem could be attributed to differences in the inoculation method employed (Ferreira et al., 1980) and differences in cultivar susceptibility.

### Sugarcane Early Responses to *S. scitamineum* Infection

Lignin and phenolic compounds were observed in cell walls at the infection site in two resistant cultivars at 24 h.a.i. but were absent in susceptible ones (**Figures 8A,C**). The induced lignification of the epidermis and parenchyma cell walls together with accumulation of phenolic compounds could provide a local barrier against *S. scitamineum* infection since fungus penetration was not observed where the tissues were lignified. However, the lignification at the infection site did not protect from *S. scitamineum* penetrating the outermost bud scales. *S. scitamineum* reportedly elicits a major accumulation of *p*-coummaric and syringic phenolic acids at 15 h.a.i. (De Armas et al., 2007). These acids are directly associated with cell wall reinforcement and could be associated with lignin deposition at the early stages of host–pathogen interaction. An increase in lignification in sclereids and tracheary elements walls of sugarcane leaves was also reported (Santiago et al., 2010b); although, leaves are not the site of infection.

Phenolic compounds located at infection sites in both resistant cultivars could be used for lignin biosynthesis together with H_2_O_2_ and oxidative enzymes (Vanholme et al., 2008; Cesarino et al., 2012). Santiago et al. (2010b) studying the effect of caffeic acid *in vitro* concluded that phenolic compounds were responsible for reduced teliospore germination. However, we observed that teliospore germination and promycelia formation were not affected by phenolics accumulation in the outermost bud scale epidermis. However, phenolic composition was not determined in the cultivars studied, suggesting a need for future biochemical analysis.

### Sugarcane Late Responses to *S. scitamineum* Infection

After infection, the fungus colonized all the tissues of the outermost bud scale in all cultivars and subsequently proceeded to the point of insertion of the scale on the stalk to get access to the developing shoot apex. Between 72 and 96 h.a.i., resistant cultivars developed a protective layer of cells with accumulation of phenolic and parietal components that appeared to be a structural and biochemical barrier against *S. scitamineum.* Callose, deposited in some cell walls of the protective layer, has been associated with plant defense response in other pathosystems (Voigt, 2014). Some protective layer cell walls exhibited a positive reaction to arabinoxylan LM11 antibody. Arabinoxylan is a hemicellulosic polysaccharide that has a cellulose-like backbone consisting of β-1,4-xylosyl residues that can bind strongly to the cellulose microfibrils by non-covalent bonds forming an intricate cross-link (Albersheim et al., 2011; Chowdhury et al., 2014). In sugarcane, it is known that arabinoxylan is strongly bound to cellulose (Souza et al., 2013). Costa et al. (2016) demonstrated that arabinoxylan is distributed in sugarcane stems in the ground parenchyma and the lignified cells from the vascular bundles. LM11 binds to unsubstituted xylan and arabinoxylans with a low degree of arabinose substitution (McCartney et al., 2005). Moreover, *p*-coumaric acid and ferulic acid was found in smut-inoculated sugarcane plantlets (De Armas et al., 2007). Both acids can be esterified to arabinoxylan (de O Buanafina, 2009). The detection of additional involvement of arabinoxylan further suggests that intricate and complex mechanisms function together to modify the cell wall composition that most likely play an important role in the prevention of further fungal colonization.

## Conclusion

In the present study, smut resistant and susceptible sugarcane genotypes were evaluated to elucidate the cell wall composition-related defense against infection by *Sporisorium scitamineum*. A plausible positive relationship between lignin deposition and initial resistance response against fungal penetration was observed. The study suggested the existence of a protective layer of cells with modified wall composition at the base of the outermost bud scale in resistant cultivars. The natural route of fungal infection in susceptible cultivars through tissues at the bud scale base was also demonstrated. Comprehensive investigations including time-course, subcellular transcriptome analysis in resistant vis-à-vis susceptible sugarcane varieties will provide detail insights into cell wall modifications toward establishment of their roles in defense mechanism of sugarcane to smut fungus.

## Author Contributions

The study was conceived and designed by JM and NB. JM conducted the microscopy analysis. JM, JH, AV, and NB were involved in field sampling and inoculation. JM, BA-d-G, JH, MV, and NB participated in writing the manuscript.

## Conflict of Interest Statement

The authors declare that the research was conducted in the absence of any commercial or financial relationships that could be construed as a potential conflict of interest.

## Footnotes

1 https://imagej.nih.gov/ij
