# Supplementary material for: Sugarcane Cell Wall-Associated Defense Responses to Infection by Sporisorium scitamineum
Source: Front Plant Sci. 2018 May 23;9:698. doi: 10.3389/fpls.2018.00698 (PMC5974332; doi:10.3389/fpls.2018.00698)
Supplement: Supplementary file 1 [file Presentation_1.PDF]

Supplementary Material

Sugarcane cell wall-associated defense responses to infection by *Sporisorium scitamineum*

João Paulo Rodrigues Marques*; Jeffrey W. Hoy; Beatriz Appezzato-da-Glória; Andrés F Gutierrez Viveros; Maria Lucia Carneiro Vieira; Niranjan Baisakh

Supplementary Figures

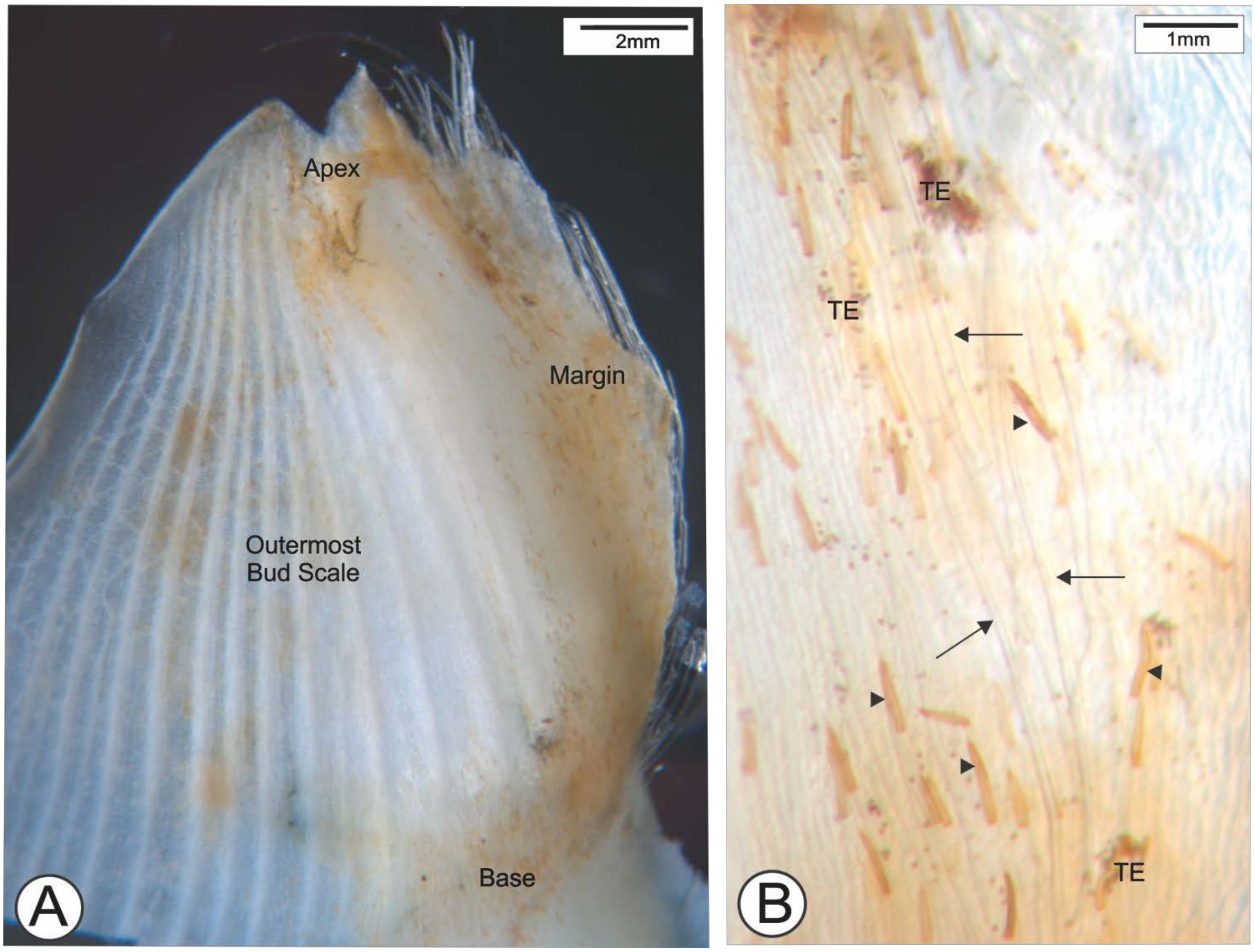

Supplementary Figure 1. Morphological features of the outermost bud scale of resistant cultivar HoCP 96-540. **A.** Overview of the scale. **B**. Detail of the margin showing two types of trichomes: a two-celled non-lignified trichome (arrowheads) and a one-celled thickened lignified trichome (arrows). TE – teliospores.

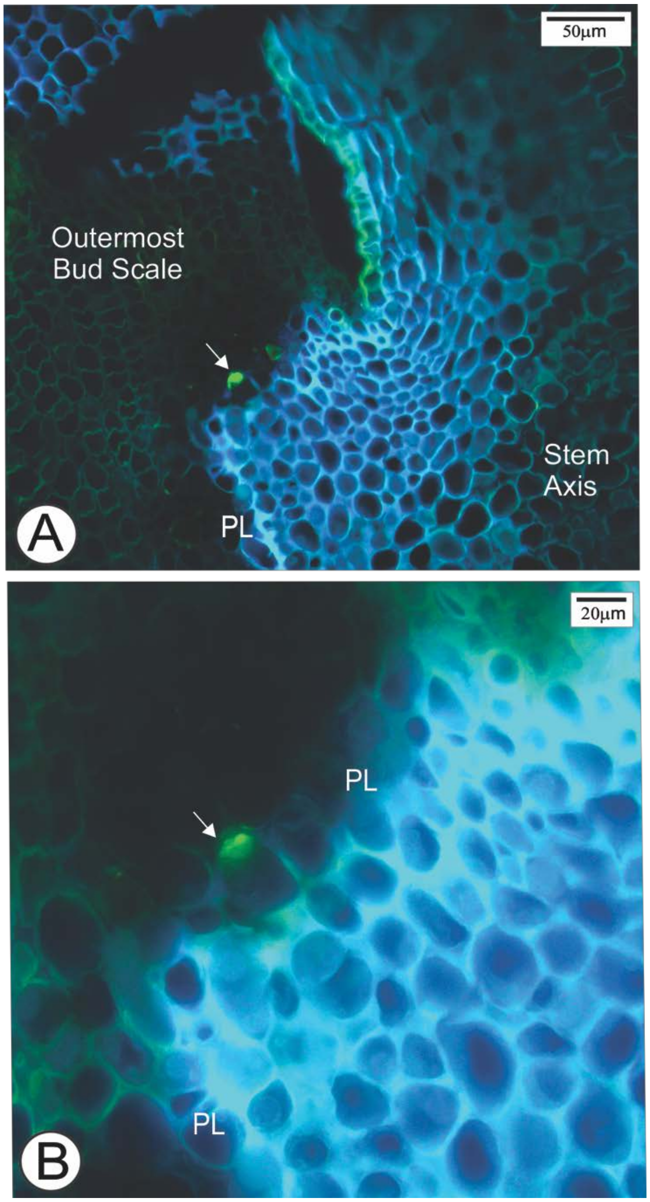

Supplementary Figure 2. Late anatomical responses of resistant cultivar LCP 85-384 to *Sporisorium scitamineum* infection at 96 hours after inoculation. **A-B.** Fluorescent images after aniline blue stain method. The protective layer (PL) presents callose deposition (arrows) in one cell. **B.** Detail of **A.**

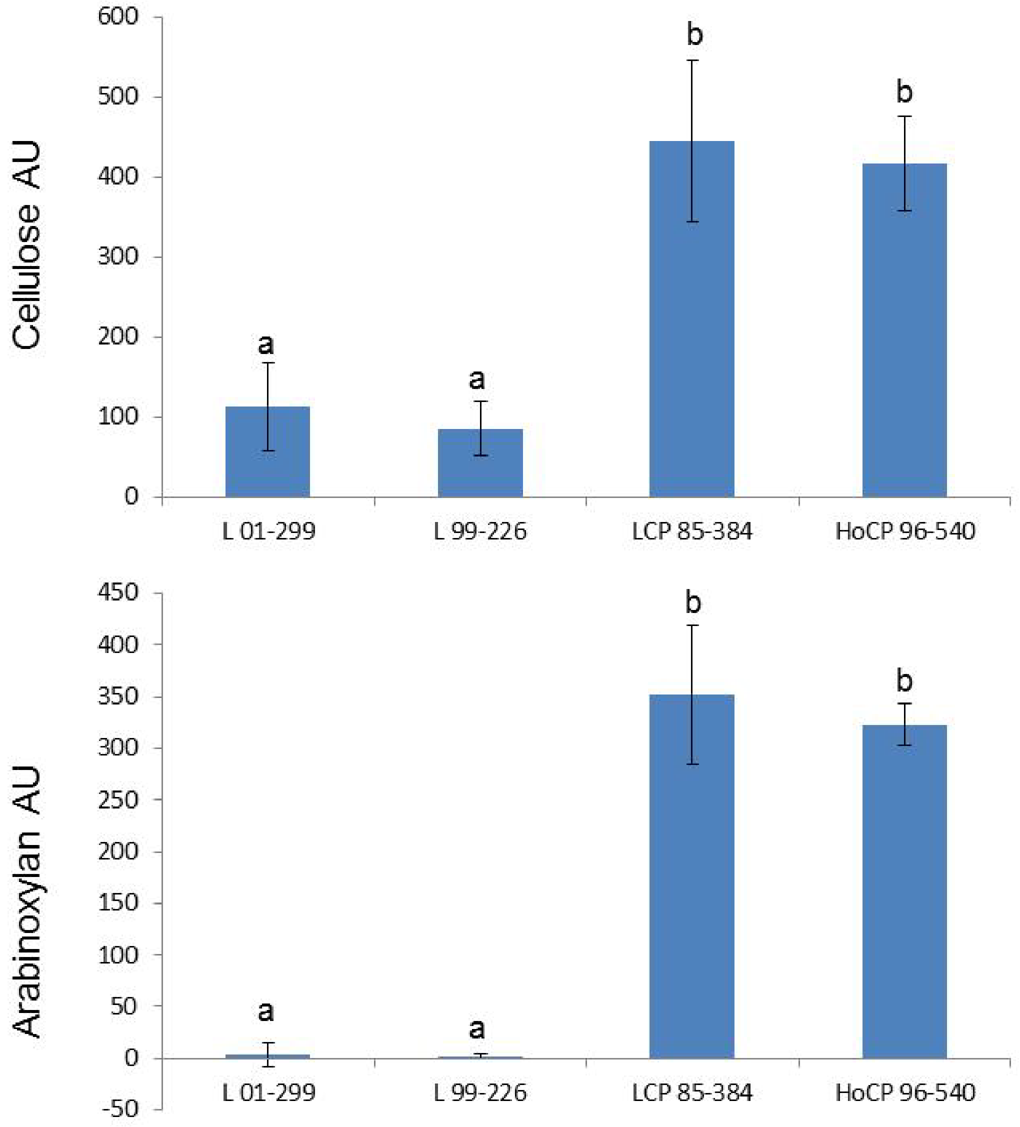

Supplementary Figure 3. Cellulose and arabinoxylan accumulation in smut resistant (LCP 85-384 and HoCP 96-540) and susceptible (L 01-299 and L 99-226) varieties of sugarcane in response to the fungal infection. AU = arbitrary unit measured as the corrected total integrated density. Bars with same letters are not statistically different ($P<0.05$; $n = 20$).
